# An Interesting Obstetrical Experience

**Published:** 1882-09-20

**Authors:** Charles H. Miller


### AN INTERESTING OBSTETRICAL EXPERIENCE.

Very early one March morning, two years ago, I was called twelve miles northwest of this city to attend Mrs. M., in labor. I had never seen Mrs. M. before, in fact had never heard of such a person before, and consequently knew nothing of her previous history, or very likely I would have invented some excuse to have remained at home. Before that Sunday had wholly passed I had been tempted fully a dozen times to commit suicide in order to escape the dreadful burden of my existence then and there.

Mrs. M. was a small, emaciated, frail, exceedingly nervous woman, about thirty years of age, and this was her second confinement. The first, I learned by snatches during the day, was attended by a physician in Illinois, from whence the family came, who devoted several days to its completion, and whose moral character, in consequence, was forever wrecked thereby.

Mrs. M. was in bed when I arrived, neatly encased in a highly embroidered night-gown, frilled and tucked, and inserted with lace, quite up to her ears, and surrounded by the entirely matronly force of the neighborhood, her large, black, searching eyes blinking like live coals, and her tongue going at the rate of one hundred and twenty strokes to the minute. The manner in which she had all of those women employed in waiting upon her would have outwitted the ingenuity of mortal man. Her pains were not worth speaking of, but as they came straggling along at the rate of one every half hour, she dextrously managed to overcome the whole loss of valuable time during such intrusions by drawing down the side of her face furthest removed from her friends, and nodding and smiling and endeavoring to keep up an unflagging interest in the general conversation with the side nearest to them. Whenever I attempted to make an examination, in order to determine, from time to time, how we were progressing toward some ultimate hope, she flinched and screwed herself in bed, and made things so decidedly uncomfortable for the entire household, that I had to desist and take my observations mentally. Moreover, such efforts always "chased away the pains," as she expressed it, and she would lie, in consequence, for hours, frequently, without even a twitch to disturb the muscles of her face. I passed the first few hours, until dawn, in fact, in desperate efforts to count the "ticks" of the clock. I was never so thankful for dawn in my life.

If there had not been some material changes in her pains about this time I truly believe I would have gone insane; but, as it was, however, these became a little more severe and prolonged, and I was thereby saved from an untimely grave. Her tongue, however, never ceased for a moment its usual rate of speed, and her perception of touch was more keenly alive than ever. Vainly, time and again, did I endeavor to administer ergot. The mere smell of the drug brought her stomach in such close proximity to her mouth, and her tongue so nearly assuming an attack of St. Vitus' dance, that I feared danger and desisted, only to resume my mental observations. By the time the child was born, near nine o'clock that night, my hands were a mass of raw flesh, from my wrists to my elbows, from the prolonged pulling they had received from time to time at the hands of that woman; my coat was nearly off my back, in pieces: part of my suspenders gone; one whole sheet used up in the same pulling; the entire neighborbood completely worn out and speechless, from sheer exhaustion, and the woman herself only the ghost of a possibility. Moreover, the child was *atelectacic;* quite dead, I thought; placed in an old shawl for ten or fifteen minutes and laid by for dead, and only discovered to possess the faintest trace of life some time afterward, *accidentally.* By throwing cold water into his face frequently and repeatedly, however; by prolonged expansion and compression of the chest; by breathing into his mouth at intervals, and by spanking him lively over various portions of his body, sufficient life was manifested during a half hour's earnest work to insure safety. The child is well and hearty to-day, and I vaccinated him this summer.—*Charles H. Miller, M.D., in Med. and Surg. Rep.*

